# Cellular therapy in mantle cell lymphoma: recommendations from the EBMT practice harmonisation and guidelines committee

**DOI:** 10.1038/s41409-026-02963-5

**Published:** 2026-07-09

**Authors:** Peter Dreger, Gloria Iacoboni, Stephen Robinson, Ali Bazarbachi, Sascha Dietrich, Toby A. Eyre, Sairah Ahmed, Timothy S. Fenske, Nilanjan Ghosh, Mehdi Hamadani, Francesco Onida, Annalisa Ruggeri, Isabel Ortega-Sanchez, Ibrahim Yakoub-Agha, Olivier Hermine

**Affiliations:** 1 Department Medicine V, University of Heidelberg, Heidelberg, Germany; 2 Department of Hematology, Vall d’Hebron University Hospital, Barcelona, Spain; 3 University Hospital Bristol NHS Foundation Trust, London, UK; 4 Bone Marrow Transplantation Program, Department of Internal Medicine, American University of Beirut, Beirut, Lebanon; 5 Department of Hematology, University Hospital Düsseldorf, Oncology and Clinical Immunology, Düsseldorf, Germany; 6 Department of Haematology, Haematology and Cancer Centre, Churchill Hospital, Oxford University Hospitals NHS Foundation Trust, Oxford, UK; 7 Department of Lymphoma and Myeloma, University of Texas MD Anderson Cancer Center, Houston, TX USA; 8 Sarah Cannon Transplant and Cell Therapy Program, Methodist Hospital, San Antonio, TX USA; 9 Atrium Health Levine Cancer Institute, Wake Forest University School of Medicine, Charlotte, NC USA; 10 Division of Hematology & Oncology, Department of Medicine, Medical College of Wisconsin, Milwaukee, WI USA; 11 Co-Chair of the Practice Harmonization and Guidelines Committee of the EBMT, Hematology and BMT Centre, ASST Fatebenefratelli-Sacco, University of Milan, Milan, Italy; 12 Co-Chair of the Practice Harmonization and Guidelines Committee of EBMT and Chair of the CTIWP of the EBMT. Hematology and BMT Unit, IRCCS San Raffaele Hospital, Milano, Italy; 13 Secretary of the Practice Harmonization and Guidelines Committee of EBMT; EBMT Medical Officer, Barcelona, Spain; 14 Chair of the EBMT Practice Harmonization and Guidelines Committee; CHU de Lille, Univ Lille, INSERM U1286, Infinite, Lille, France; 15 Department of Adult Hematology, Necker Hospital, Imagine Institute, Université De Paris-cité, Assistance Publique Des Hôpitaux De Paris, Paris, France

**Keywords:** Stem-cell therapies, B-cell lymphoma

## Abstract

Cellular therapies, namely autologous hematopoietic cell transplantation (autoHCT), allogeneic HCT (alloHCT) and more recently, chimeric antigen receptor T-cell therapies (CART), play a major role in state-of-the-art treatment of mantle cell lymphoma (MCL). With numerous therapeutic innovations currently entering the MCL treatment landscape, guidance for indication, sequencing and application of cellular therapies is of key importance. To address this practical need, the European Society for Blood and Marrow Transplantation (EBMT) Practice Harmonisation and Guidelines Committee convened an international expert meeting in Berlin on Sept 29/30, 2025. EBMT provided funding and logistical support. Two appointed chairpersons invited experts in MCL and cellular therapy, who completed three work packages devoted to the three types of cellular therapy. A literature search was performed beforehand and suitable published evidence was considered collectively in structured discussions and consensus-building exercises. The recommendations developed as a result of this process provide a sound basis for practical counseling and decision-making in the care of patients with treatment-naïve and relapsed/refractory MCL.

## Questions asked/issues to be addressed

In these recommendations, the following questions are addressed regarding CAR T-cell therapy (CART), autologous and allogeneic hematopoietic cell transplantation (auto-HCT and allo-HCT) in mantle cell lymphoma (MCL):

- How to categorize MCL risk
- CAR T-cell therapy
  1. Who are candidates for CART?
  2. What needs to be considered in terms of holding and bridging therapies prior to CART in patients with MCL?
  3. What needs to be considered regarding post-CART management?
- Auto-HCT
  1. Who should be considered for auto-HCT as part of first-line treatment?
  2. Is there a role for auto-HCT in the MCL salvage setting?
  3. What are the requirements for patient eligibility for auto-HCT in MCL?
  4. What needs to be considered regarding post-transplant management, including consolidation or maintenance therapy?
- Allo-HCT
  1. Where is the place of allo-HCT in the MCL treatment algorithm?
  2. What are the requirements for patient eligibility for allo-HCT in MCL?
  3. How to perform allo-HCT in patients with MCL?
  4. How to prevent and manage post-transplant relapse in MCL patients.

## Current state of the art

Mantle cell lymphoma (MCL) is a B-cell lymphoma with an unfavourable natural course with conventional chemoimmunotherapy. Besides high-dose ara-C- and rituximab-based chemoimmunotherapy, cellular therapies, namely autologous hematopoietic cell transplantation (auto-HCT), allogeneic HCT (allo-HCT) and, more recently, CAR T-cell therapies (CART), have contributed to improving the prognosis of MCL significantly and play a major role in modern MCL treatment algorithms. A variety of guidelines and expert consensus papers, including a few from this organization on the use of cellular therapies in MCL management, have been published to date [1–4]. The dynamic evolution of the MCL treatment landscape, as well as of cellular therapy technologies, requires timely updates of practice recommendations regarding the optimum use of cellular therapy in MCL.

Therefore, the EBMT Practice Harmonization & Guidelines Committee and the EBMT Lymphoma Working Party of EBMT decided to include cellular therapy in MCL in the 2025 edition of the EBMT Best Practice Recommendations Initiative.

## Methodology

In May 2025, the EBMT Lymphoma Working Party appointed two project leaders (PD and OH) with the task of developing consensus-based recommendations on cellular therapy in MCL within the 3^rd^ edition of the EBMT Best Practice Recommendations Initiative. A panel of seven EBMT-affiliated experts and four external experts were selected based on their professional expertise and willingness to contribute actively to the project and serve as co-authors of this work. The methodological framework of the project adhered to the standards and methods of the EBMT Practice Harmonisation and Guidelines Initiative [5]. Procedural details and project objectives were proposed by the project leaders and endorsed by the selected experts, including the Lymphoma Working Party chairperson and the EBMT Practice Harmonisation and Guidelines Committee. The project was structured in three major work packages (CART, auto-, allo-HCT), each consisting of several subtopics addressing pre-specified key questions (Appendix). The final list of key questions and topics was collectively agreed upon by all participating experts. For each subtopic, leads were assigned from the expert panel responsible for collecting and structuring the published evidence for their respective task.

A 2-day workshop was conducted in Berlin on Sept 29 and 30, 2025, with the participation of 5 EBMT-affiliated experts on-site (PD, AB, GI, OH) and online (SR), respectively. Two European experts could not attend due to logistical reasons (SD, TE). For each thematic area, compiled evidence elaborated by the topic leads was presented, discussed and converted into provisional draft recommendations. Given the paucity of randomised trial data and the inadequate evidence base for many scenarios, recommendations were not formally graded. Instead, a structured consensus approach was used, incorporating iterative expert discussions. The draft recommendations developed as a result of the workshop were circulated and extensively discussed among all EBMT and external experts. The final recommendations and the manuscript were validated and endorsed by the whole panel. The EBMT provided logistical support for the whole process, including the workshop.

## EBMT recommendations

### Risk categorization for MCL

Several biological and clinical risk factors which are associated with poor response to chemoimmunotherapy and auto-HCT and reduced efficacy of Bruton tyrosine kinase inhibitors (BTKi) have been identified in MCL. These can be used to define risk categories in order to enable risk-adapted allocation and sequencing of the various cellular therapies [6–8].

At baseline, mutations of the *TP53* gene indicate relative refractoriness to chemoimmunotherapy, resulting in a significantly increased mortality risk with CIT/auto-HCT-based treatment strategies [9, 10]. In addition, TP53 deletions, p53 overexpression on immunohistochemistry ( > 50%), a high KI-67 proliferation index ( > 30%) and a high MCL International Prognostic Index including Ki-67 (MIPI-c) can be used to define *high-risk MCL,* although their adverse impact on outcomes is weaker [9, 11–14]. Because it is superseded by KI-67, blastoid or pleomorphic histology is only considered as high-risk defining if KI-67 information is not available [12, 14]. Biological high-risk features also affect the outcome of BTKi-based induction and salvage therapies, albeit to a lesser extent [15–17].

In patients who experienced first-line treatment failure, the interval between treatment initiation and failure event represents an additional important risk factor, as progression or relapse of treated MCL within 24 months (POD24) is associated with substantially reduced survival independent of the aforementioned biological risk factors [18–20]. Recent data also suggest that failure of MRD clearance at 10-6 level using the clonoSEQ assay [21] after consolidation with auto-HCT during induction therapy may be associated with an impaired prognosis [22].

By contrast, patients with non-nodal MCL and those with asymptomatic classical nodal MCL and low proliferation index are considered to have *low-risk MCL* [23, 24]. These patients typically follow different management algorithms, currently do not need any cellular therapy in the first line and will not be further addressed in this paper.

All patients not falling into these two categories will be defined as having *standard-risk MCL*.

**Panel 1. MCL risk categorization**

- **High-risk**
  - At the start of 1L treatment (or evolving during disease course):
    - TP53 mutation (strong effect, median OS <3y with CIT +/– autoHCT)
    - TP53 deletion, p53 expression >50% (immunohistochemistry), Ki-67 >30%, MIPI-c high, blastoid/pleomorphic variant (in the absence of Ki-67 information) (moderate effect, median OS about 4-5y).
  - At the end of induction after autoHCT, the provisional criterion:
    - MRD persistence (clonoSEQ 10^–6^ level) (strong effect, but still limited evidence).
  - At first relapse/progression:
    - Progression of disease within 24 months of treatment initiation: POD24 (strong effect, median OS from relapse <1y)
- **Standard-risk**: all other patients.
- **Low-risk:** indolent MCL (non-nodal without adverse genetic features or nodal without symptoms, high tumour burden, high Ki-67, or blastoid morphology).

### CAR T-cell therapy

#### CART indications: first-line treatment

Despite reduced efficacy of standard CIT/auto-HCT-based treatment approaches in patients with high-risk MCL, modern BTKi-containing first-line regimens can provide durable disease control in the majority of patients with high-risk features [25, 26]. Therefore, CARTs as part of first-line therapy in high-risk MCL have to be considered as experimental and should be performed only within prospective clinical trials, such as CARMAN (NCT064822684) or BMT CTN 2502 study (personal communication M. Hamadani) [2, 3, 27]. There is no role for CART in the first-line therapy of standard-risk MCL.

#### CART indications: relapsed and refractory disease

Brexucabtagene autoleucel (Brexucel) and, recently, Lisocabtagene maraleucel (Lisocel) are currently the only commercially available CART therapies for MCL in Europe, both approved for patients who have received 2 lines of systemic treatment, including a BTKi. Nevertheless, in the absence of effective standard therapies for patients who experience disease progression following BTKi-containing frontline treatment, CAR T-cell therapy may be considered in the second-line setting for this high-risk group when enrolment in a CAR T–based clinical trial is not feasible. Despite favourable trial data [28], patients who are BTKi-naïve at second line have currently no indication for CART in Europe outside of a clinical trial, irrespective of their risk category.

Beyond the second line, CD19 CARTs are the treatment of choice for all CART-naïve patients. While there is no evidence supporting repeat CD19 CART therapy in patients with MCL, the use of CARTs employing alternative targets and co-stimulatory elements is an option, to date only within clinical trials [29]. A prior alloHCT is not a contraindication for CART therapy [30].

**Panel 2. Recommendations for CART indications**

- **First-line**
- High-risk MCL: only in trials.
- Standard-risk MCL: no role.
- **Second-line**
- BTKi-naïve: no role outside of clinical trials.
- BTKi-exposed: clinical option, especially in those patients who progressed while still on BTKi, or within a clinical trial.
- **Beyond second-line**
- CART-naïve: standard of care.
- CART-exposed: no role for repeat CD19 CART. Consider an alternative CART in clinical trials.

#### Eligibility for CART treatment

Similar to LBCL, there is no strict upper age limit for CART treatment in MCL [31, 32]. Instead, it appears to be reasonable to follow published algorithms for CD19 CARTs, suggesting that the key patient-related factors of age, performance status and comorbidity collectively define individual CART eligibility [3, 33]. A careful assessment of performance status as a composite indicator of age, comorbidity and tumour biology-related impairment is warranted [34, 35]. Whether formal geriatric assessment in older patients may help in defining CART eligibility remains to be shown [36, 37].

#### Tumour-related baseline prognostic factors for CART treatment

The evidence that CAR T-cell therapy can overcome the adverse impact of high-risk MCL-defining features is still conflicting. Where investigated, response rates do not appear to be significantly affected by TP53 alterations, KI-67 index, or blastoid morphology [38, 39]. However, responses seem to be less durable in the presence of high-risk features in some, but not all, series reported. More consistent evidence supports the notion that patients with short remission duration following first-line (POD24) or subsequent therapies have an inferior outcome after brexucel [39–41]. Prognostic parameters identified in individual retrospective studies are described in Table 1. Among these are indicators of tumour activity (LDH, bulky disease, extra-nodal involvement) and inflammation (CRP, ferritin), which are well- established risk factors of CART outcomes in LBCL [42], but need to be further validated for MCL.

**Table 1:** Real-world series on brexu-cel for MCL: Adverse factors for progression-free survival.

	UK [40].	DESCAR-T [46].	SIE [101].	DRST/GLA/ SAKK [41].	US CART consortium [39].	CIBMTR [32, 35].
*N*	83	152	106	113	168	476
**Patient-related**						
Age (higher)	+	n.a.	n.a.	–	–	–
PS (poorer)	–	n.a.	n.a.	n.a.	+	++
**Disease-related**						
Ki-67 ≥ 30%	–	n.a.	n.a.	–	+	n.a.
Blastoid/pleomorph.	n.a.	n.a.	+	–	+	n.a.
TP53abn	–	n.a.		–	+	n.a.
POD24	+	n.a.	–	++^a^	+	n.a.
Refractory @ LD		+	n.a.	+	–	n.a.
Other	Male sex, bulk >5 cm, extra-nodal sites >2	CRP ↑ , LDH ↑	LDH ↑, bulk			
**Pretreatment-related**						
Pretreatment lines	–	n.a.	n.a.	–		+
Prior bendamustine	–	n.a.	n.a.	+	+	–
Prior BTKi ref.	n.a.	n.a.	+	n.a.	–	n.a.
Prior HCT	–	n.a.	n.a.	–	n.a.	–

^a^POD12.

*BTKi* Bruton’s tyrosine kinase inhibitor, *HCT* hematopoietic cell transplantation, *LD* lymphodepletion, *n.a.* not available, *POD24* progression of disease within 24 months from start of first-line therapy, *PS* performance status.

Limited evidence is available for CART therapy in MCL patients with CNS involvement. While response rates appear to be as encouraging as in patients without CNS disease, short response duration is a concern, especially in patients with active CNS disease at lymphodepletion [43].

**Panel 3. Recommendations for CART eligibility and baseline outcome predictors**

- **CART eligibility**

- There are no strictly defined age limits or single comorbidities that preclude CART eligibility in MCL.
- Performance status, comorbidity and age collectively define individual CART eligibility.
- **Tumour-related outcome predictors**
- MCL high-risk criteria may adversely affect response duration after CART therapy.
- LBCL-typical unfavourable biological parameters, such as high tumour volume, tumour proliferative activity and inflammation markers, still need to be validated in MCL.
- **Prognostic scores**
- CAR-HEMATOTOX is the only prognostic score validated for MCL CART treatments to date.

#### Prognostic scores

Similar to CART use in LBCL and multiple myeloma, the CAR-HEMATOTOX score (which takes into account pre-lymphodepletion cytopenias, ferritin and CRP levels) predicts the risks of prolonged cytopenias, severe infections, NRM and overall mortality, also after brexucel treatment of MCL [44]. Other prognostic scores effective in LBCL and/or multiple myeloma CART settings, such as the EASIX [45], still need to be validated in MCL.

#### Holding and bridging

Holding therapies are defined as those administered between indication for CART and leukapheresis and bridging therapies as those given between leukapheresis and CART infusion. Except for the French and German real-world studies [41, 46] the beneficial effects of holding/bridging therapy on survival outcomes have not been demonstrated in other major MCL CART studies published to date [38–40, 47]. The depth of disease control/response with bridging to achieve the optimum outcome with CART in MCL is currently unclear. Nevertheless, there may be medical needs requiring immediate intervention in the form of holding/bridging therapies, such as symptom control, keeping the patient stable until CART infusion, and mitigating pro-inflammatory effects of active lymphoma. There is no need to achieve an objective response as a prerequisite for proceeding to CART administration, even in case of CNS involvement, as long as there is no aggressively proliferating disease with large tumour volume and/or heavy leukemic expansion. Thus, the treatment goal of holding and bridging is avoiding massive disease progression and providing symptom relief during CART manufacturing rather than achieving a state of minimal tumour load prior to lymphodepletion [33]. In addition, the expected duration of CART production time influences the decision to consider holding/bridging therapies.

**Fig. 1:**
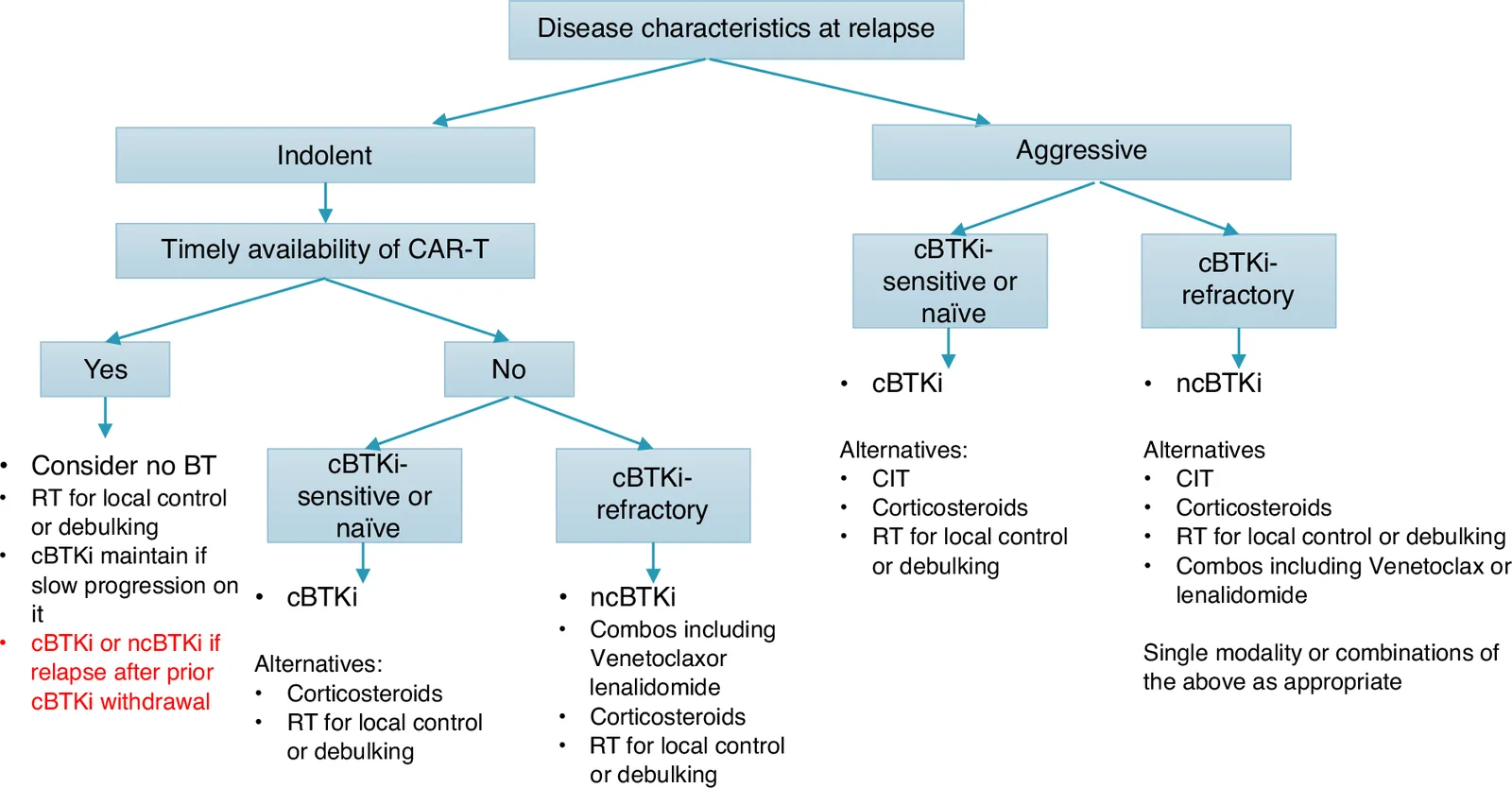
Algorithm for bridging therapy in MCL. (BT bridging therapy, cBTKi covalent Bruton’s tyrosine kinase inhibitor, CIT chemoimmunotherapy, ncBTKi non-covalent Bruton’s tyrosine kinase inhibitor, RT radiotherapy, PS performance status).

There is no standard approach to holding/bridging in MCL; the choice is based on the aggressiveness of the disease, treatment history (including sensitivity to prior agents), age, comorbidities, performance status, expected product turnaround time, and the need for systemic versus local control. In-label holding/bridging options in covalent BTKi-refractory patients include pirtobrutinib and MCL-effective chemoimmunotherapy, with the exception that bendamustine-containing regimens should not be used for holding because of their severe lymphodepleting effects [39, 48–50]. In addition, it is still not settled whether CD19-targeting agents (which are currently not approved in MCL) could affect the outcome of subsequent CD19-directed CART therapy [33, 51]. Radiotherapy should be considered for single symptomatic or bulky sites. A suggested algorithm for holding/bridging is shown in Fig. 1. Other, still off-label bridging options comprise combinations of venetoclax with covalent BTKi and CD20 antibodies, and bispecific antibodies [52–54].

If the patient is on BTKi and still sensitive (ongoing response or only slow progression), BTKi should be maintained through leukapheresis until lymphodepletion to avoid tumour flare and take advantage of presumptive T-cell fitness-enhancing effects [55] and improvement of the myeloid suppressive environment. This hypothesis has been tested with ibrutinib in the TARMAC study, with promising results even in covalent BTKi-resistant patients [56].

**Panel 4. Recommendations for holding and bridging strategies**

-  **Treatment goal**

- Treatment goal of holding/bridging is symptom control and keeping the patient stable rather than achieving minimal tumour load prior to CART.
- Avoid delaying CART if already available unless there is massive disease progression.
- **Selection of holding/bridging strategy**
- The decision to use holding/bridging therapies is based on disease biology, tumour kinetics and the presence of symptoms.
- There is no standard approach for holding/bridging in MCL; the choice is based on the aggressiveness of the disease, prior treatments, age, comorbidities, available options, expected turnaround and the need for systemic vs local disease control.
- If the patient is on BTKi, BTKi should be maintained through leukapheresis.
- Bendamustine should be avoided for holding therapies.

#### Product selection

Brexucel and, since December 2025, lisocel are both approved by the EMA for the treatment of adult patients with relapsed or refractory MCL after at least two lines of systemic therapy, including a BTKi. According to the registration trials, lisocel is associated with less neurotoxicity than brexucel, which could make this product more suitable, particularly for older and comorbid patients. However, if this translates into a better safety profile in terms of ICAHT, infection risk, and NRM still needs to be shown. Although both CARTs achieved high CR rates in their respective approval trials (brexucel 68%, lisocel 75%) [38, 49], responses were not stable (median duration of response with brexucel 28 months, lisocel 16 months) without a clear-cut plateau, implying that evidence of the curative potential of CART therapy in MCL is still lacking. European real-world data on brexucel are in line with the results of the brexucel approval trial ZUMA-2 [3]. Currently, there is no conclusive data showing the efficacy advantages of one of these two products over the other, with the caveat that the body of evidence available for lisocel in this indication is still very limited. Moreover, the published turnaround times (from leukapheresis to product delivery) are shorter for brexucel than for lisocel. These issues need to be weighed against the more favourable toxicity profile of lisocel on a patient-by-patient basis.

#### CART administration

As there is no specific procedure for product administration in patients with MCL, we refer to the EBMT general CART best practice recommendation [57]. and the authorized product information of the two labelled CARTs (Tecartus, INN-brexucabtagene autoleucel) (Breyanzi, INN-lisocabtagene maraleucel).

#### Post-infusion management

Although NRM appears to be higher after CART treatment in MCL compared to other indications, with infections as the leading cause of death [58], an MCL-specific pattern of infections or complications that suggests modifications of post-CART supportive care could not be identified yet. Therefore, the generally recommended measures apply also to MCL [57]. Preliminary data suggest a beneficial role of immunoglobulin replacement after brexucel for MCL [41], but current evidence is still insufficient to support prophylactic immunoglobulin administration policies.

Assessment of measurable residual disease by molecular detection methods following CART therapy in MCL may have prognostic implications [22, 49] but lacks prospective validation in this setting, without data supporting a benefit of pre-emptive interventions. The same accounts for post-CART PET-CT surveillance [59, 60]. PET-CT may be useful, however, to select an appropriate site for biopsy in those patients who fail to achieve morphologic CR beyond 3 months after CART infusion.

There is currently no role for post-CART consolidation or maintenance, e.g. with CD20 antibodies or BTKi, outside of clinical trials.

**Panel 5. Recommendations for post-CART management**

- **Supportive care**

- Routine supportive care policies should follow general post-CART management guidelines.
- **Response surveillance**
- There is currently no established role for post-CART MRD monitoring and/or PET-CT surveillance as part of the clinical routine.
- Patients who do not achieve a CR as the best response to CART must be considered as treatment failures and are candidates for biopsy, ideally guided by PET imaging.
- **Maintenance**
- There is currently no role for post-CART consolidation or maintenance, e.g. with CD20 antibodies or BTKi, outside of clinical trials.

### Autologous hematopoietic cell transplantation

#### Auto-HCT indications: first-line treatment

Since its introduction to routine clinical care more than 20 years ago, high-dose ara-C-based chemoimmunotherapy with auto-HCT consolidation has been considered as the backbone of first-line therapy in younger patients ( ≤ 65 years) [2, 61–63]. This paradigm is now challenged by the advent of BTKi in MCL upfront treatment. The *TRIANGLE* trial compared standard frontline therapy (ara-c-based chemoimmunotherapy followed by auto-HCT consolidation and rituximab maintenance) without (A) or with ibrutinib (A + I) with a third arm with ibrutinib but without auto-HCT (I) and could not show an additional benefit of auto-HCT consolidation in terms of failure-free survival when ibrutinib is given as part of induction and fixed duration maintenance therapy [64]. However, the follow-up of *TRIANGLE* is still too short to exclude the inferiority of I vs A + I after rituximab maintenance has ended. Indeed, an unplanned post-hoc analysis of the *TRIANGLE* trial showed a non-significant PFS benefit at the 4-year landmark (90%; 95% CI 85–96% vs 85%; 95% CI 78–91%) in favour of A + I over I for those patients who had received rituximab maintenance [65]. In addition, subgroup analyses from the *TRIANGLE* trial showed a non-significant trend toward failure-free survival benefit for A + I in patients with high-risk MCL [64]. However, ibrutinib maintenance following auto-HCT provided substantially more non-fatal toxicity than ibrutinib maintenance without auto-HCT.

On the other hand, in patients with favourable IGHV families, A + I did not provide additional benefit compared to A in *TRIANGLE* [66]. Finally, the added value of auto-HCT in patients who achieve CR with MRD clearance at the 10-6 level after induction with or without BTKi is questioned by the randomized ECOG 4151 trial, where achievement of MRD-negativity after induction (regardless of induction regimen) identified a group that appears to derive little benefit from auto-HCT, provided that rituximab maintenance for 3 years is given subsequently [22].

Taken together, the results of both the *TRIANGLE* and the ECOG 4151 trials, as well as some real-world data [67], provide evidence that consolidative auto-HCT as part of first-line therapy can be safely omitted at least in standard risk MCL if BTKi are part of induction and/or MRD clearance at the 10-6 level is achieved. As the long-term implications of this strategy are still not settled, the pros and cons of auto-HCT in this setting have to be discussed in depth with the patient before decision-making.

#### Auto-HCT indications: relapsed and refractory disease

With respect to relapsed and refractory MCL, only a few transplant-eligible patients will currently arrive HCT-naïve in the salvage setting. This situation may change with 1 L auto-HCT being increasingly replaced by BTKi. However, the majority of early relapses on BTKi-based 1 L regimens will be characterized by high-risk features and the feasibility and efficacy of autologous transplantation in BTKi-refractory patients are largely unclear.

**Panel 6. Recommendations for indications of consolidative auto-HCT in MCL**

- **First-line, without BTKi**

- TP53 mutations present: only in trials.
- Other high-risk and standard-risk: auto-HCT consolidation is recommended. Consider omission if MRD is undetectable at the 10^–6^ level (clonoSEQ) after induction.
- **First-line, with BTKi**
- High-risk: auto-HCT consolidation should be considered, especially if MRD-positive.
- Standard-risk: omission of auto-HCT should be considered, especially if MRD-negative (10^–6^) after induction. Discuss with the patient that the possible late benefits of auto-HCT have to be weighed against its toxicity.
- **Second-line and beyond**
- There is no evidence supporting a role for auto-HCT in the salvage setting in the current MCL treatment landscape.

#### Eligibility for auto-HCT

Although age (cut-off 65 or 70 years) has been used as a factor defining auto-HCT-ineligibility in some CART trials [68, 69] there is no strict upper age limit for auto-HCT in MCL or in general. Jantunen et al. compared patients autografted for MCL who were aged between 65 and 73 years with younger patients and found similar outcomes [70]. However, all high-level randomized trial-based evidence on auto-HCT consolidation in MCL has been obtained in patients <66 years [25, 63, 71]. Patients older than 65 years are heavily underrepresented in real-world analyses on auto-HCT in MCL (or any other auto-HCT indication) and data on auto-HCT beyond the 75-year landmark are very limited. Therefore, it can be stated that auto-HCT can be safely performed in patients with MCL aged 65 years or older, but the proportion of eligible patients reduces with increasing age and the benefit of auto-HCT in this age group is only poorly documented. Patient suitability should be assessed by a clinician experienced in auto-HCT, and age, along with performance status, comorbidities and alternative treatment options should be considered.

Patients with disease unresponsive to standard induction chemoimmunotherapy have been generally excluded from the pivotal trials establishing the role of consolidative auto-HCT in MCL. Nevertheless, extrapolation from other lymphoma entities, biological plausibility and preliminary retrospective data [70, 72] support the established consensus that auto-HCT should not be performed in patients with refractory MCL [3, 27, 73].

**Panel 7. Recommendations for auto-HCT eligibility in MCL**

- **Patient-related: age**
- There is no high-level evidence supporting the use of consolidative auto-HCT as part of first-line MCL therapy in patients older than 65 years.
- Nonetheless, auto-HCT can be safely performed in eligible MCL patients older than 65 years, but the benefit of auto-HCT in this age group is questionable.
- **Patient-related: general**
- Patient suitability should be assessed by a clinician experienced in auto-HCT.
- Assessment should consider age, performance status, comorbidities and alternative treatment options.
- **Disease status**
- Auto-HCT is not recommended in patients not achieve at least a PR on induction therapy.

#### How to perform auto-HCT

BEAM (carmustine, etoposide, ara-C, melphalan) is considered the standard high-dose regimen for auto-HCT in MCL. Myeloablative doses of total body irradiation (TBI) or busulfan in combination with cyclophosphamide (BuCy) can be an alternative to BEAM in case of carmustine or melphalan shortage [72, 74]. No other high-dose regimen has been adequately tested or can be recommended outside of clinical trials in this setting. There is no role for in-vitro or in-vivo purging of the graft in auto-HCT in patients with MCL in the rituximab era [1].

#### Post-auto-HCT management

The *LYMA* trial established rituximab maintenance (375 mg/m^2^ every 2 months for 3 years) as standard after 1st-line consolidative auto-HCT in MCL, providing significantly superior progression-free survival (hazard ratio (HR) 0.36; 95% CI 0.23–0.56) and numerically superior overall survival (HR 0.63; 95% CI 0.37–1.08) compared to no maintenance [75]. Preliminary data from *TRIANGLE* suggest that the benefit of rituximab maintenance persists even when ibrutinib is part of frontline therapy 11684.

Although MRD monitoring by allele-specific quantitative PCR (qPCR) can help to identify patients at increased risk of relapse during the post-transplant course {7404}, the benefit of MRD-guided pre-emptive interventions, such as rituximab treatment, is uncertain [76].

Posttransplant surveillance, supportive care and infection prophylaxis should follow general guidelines [77].

**Panel 8. Recommendations for post-auto-HCT care**

- **Maintenance**
- Rituximab maintenance (for 3 years) is recommended after first-line consolidative auto-HCT for MCL, irrespective of concurrent BTKi maintenance.
- **MRD monitoring**
- Irrespective of the technology used, there is currently no established role for post-auto-HCT MRD monitoring as part of the clinical routine in MCL.

### Allogeneic hematopoietic cell transplantation

#### Allo-HCT indications: first-line treatment

Given its morbidity and mortality risks on the one hand and the excellent outlook with modern first-line therapies on the other hand, upfront allo-HCT has no role in current MCL treatment algorithms [2, 3, 27].

#### Allo-HCT indications: relapsed and refractory disease

The evidence for allo-HCT in BTKi-pretreated patients is limited [78, 79]. In the only larger analysis to date, the EBMT reported 12-month estimates for OS, PFS, NRM and relapse incidence (REL) of 66%, 56%, 19% and 26%, respectively, in 272 patients who underwent allo-HCT for MCL after BTKi exposure [80]. This appears to be comparable, however, to the 12-month OS, PFS, NRM and REL rates of 62%, 51%, 24% and 25%, respectively, observed in a previous EBMT study including 324 patients from the pre-BTKi era [81]. Thus, in settings where CARTs are not available, allo-HCT could be considered for second-line consolidation in salvage-sensitive patients who progressed on BTKi-based first-line therapy or do not achieve CR on second-line BTKi at the 6-month landmark [2, 3, 27]. The role of a consolidative allo-HCT in patients achieving a CR to second-line BTKI is uncertain but may be considered in high-risk patients where no CART is available and preferably within the context of a clinical study.

The ZUMA-2 trial population had a significantly better OS when matched 1:1 to a cohort of BTKi-exposed allo-HCT recipients from the EBMT registry (3-year rate 57% vs 45%, median follow-up 36 months) [80]. This was largely due to significantly lower NRM in the ZUMA-2 cohort (3.6% vs 21.2% at 12 months), supporting the notion that CART should be preferred over allo-HCT in BTKi-refractory patients even if they are allo-HCT eligible.

**Fig. 2:**
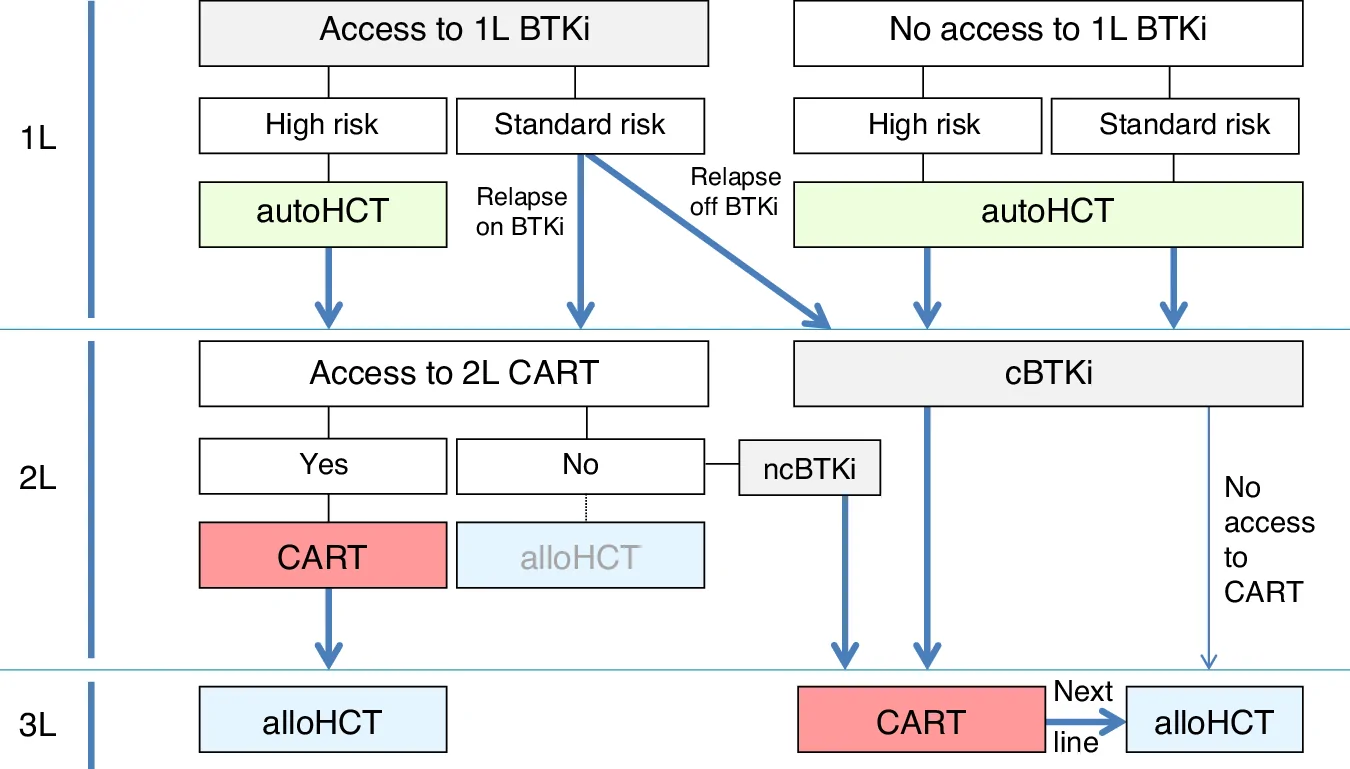
Algorithm for sequencing cellular therapies in MCL. (Allo-HCT allogeneic hematopoietic cell transplantation, Auto-HCT autologous hematopoietic cell transplantation, BTKi Bruton’s tyrosine kinase inhibitor, CIT chemoimmunotherapy, cBTKi covalent BTKi, ncBTKi non-covalent BTKi, RT radiotherapy, PS performance status).

This moves allo-HCT to the next line after CART [2, 3, 7] (Figure 2). Unfortunately, published evidence on the feasibility and efficacy of allo-HCT in this setting in MCL is extremely sparse. An American real-world analysis investigated the outcome of 135 patients with MCL who relapsed or were refractory to CD19-directed CART therapy. With a median OS of less than 6 months, the outcome was poor in this population, in particular for those patients who had failed CART within the first 3 months. However, of the 8 patients who managed to proceed to consolidative allo-HCT after successful salvage therapies, 6 were alive and disease-free at a median follow-up of nine months after transplantation [82]. This small piece of evidence is the first supporting the concept of rescuing CART failures in MCL with allo-HCT, similar to LBCL [83, 84].

**Panel 9. Recommendations for allo-HCT indications**

- **First-line**
- No role outside of clinical trials.
- **Second-line**
- BTKi-naïve: no role.
- Progressed on BTKi-based first-line therapy or no CR on second-line BTKi; no access to CART: consider allo-HCT as a consolidation option in salvage-sensitive patients.
- High-risk MCL; CR on second-line BTKi; no access to CART.
- **Next line after CART**
- Clinical option in salvage-sensitive patients.

#### Eligibility for allo-HCT

The same principles delineated for auto-HCT also apply to allo-HCT eligibility: The age groups beyond 65 years are heavily underrepresented in the published evidence and there is virtually no information on allo-HCT in patients with MCL beyond the 75-year landmark. Both EBMT and CIBMTR have investigated the outcome of allo-HCT in elderly patients ( ≥ 65 years old) with NHL, including MCL and observed a relatively high NRM (about 40% at 4 years) despite using reduced-intensity conditioning (RIC) in the vast majority of patients, whereas the incidence of relapse did not appear to be increased compared to younger cohorts [85, 86]. Similarly, in a more recent EBMT analysis restricted to patients with MCL, older age ( > 60 years) was associated with an increased risk for non-relapse and overall mortality (HR 4.13, 95% CI 0.97–17.6; HR 3.0, 95% CI 1.18–7.61) [80]. This indicates that allo-HCT in MCL beyond the 65-year landmark is feasible, but patients need to be carefully assessed by a clinician experienced in allo-HCT, considering comorbidities, performance status and biological age.

While allo-HCT seems to overcome the adverse impact of high-risk features, such as TP53 mutations [87] and POD24 [18], disease status at transplantation has a major effect on allo-HCT outcome. In the 2 aforementioned EBMT studies from the pre-BTKi and the BTKi era, respectively, patients undergoing allo-HCT with refractory MCL had significantly inferior outcomes compared to those with responsive disease [80, 81]. However, with 3-year OS rates between 25% and 30%, even the patients proceeding to transplant with stable or progressive lymphoma seemed to gain some benefit. Similar survival probabilities were reported from a CIBMTR study focusing on allo-HCT in patients with refractory MCL [88].

**Panel 10. Recommendations for allo-HCT eligibility in MCL**

- **Patient-related**

- Patient suitability should be assessed by a clinician experienced in allo-HCT.
- Assessment should consider age, performance status, comorbidities and alternative treatment options.
- Allo-HCT can be performed in patients with MCL aged 65 years or older, but the increasing risk of NRM should be considered.
- There is no evidence supporting allo-HCT in MCL beyond the 75-year landmark.
- **Disease-related**
- The presence of HR criteria should not preclude allo-HCT
- Although unresponsive disease strongly affects outcome, allo-HCT can provide durable disease control in a minority of patients with refractory MCL

#### Bridging to allo-HCT

In contrast to the CART setting, the treatment goal of bridging therapies for allo-HCT should be the induction of a robust response and minimal tumour load for enabling disease control until the GVL becomes effective. Suggested regimens are the same as discussed for bridging in CARTs, with the exceptions that bendamustine may be used pre-alloHCT and a wash-out period between the last dose of bispecific antibodies and transplantation to avoid increasing the GVHD risk might be advisable [89]. Generally, bridging to allo-HCT has to be designed individually, considering the approved and experimental treatment options left and their individual safety/efficacy profile.

#### Conditioning for allo-HCT

Several registry studies and one systematic meta-analysis demonstrate that in MCL allotransplants, outcomes with RIC are at least equivalent to those obtained with myeloablative conditioning [90–92]. even in patients with refractory disease [88]. This notion is also supported by a large registry analysis showing that the most intensive conditioning regimen had the worst outcome among 1823 lymphoma RIC transplants [93]. Accordingly, RIC is the preferred choice, thereby allowing wider application of allo-HCT in the older and more vulnerable target population of patients affected with MCL [1, 3]. There is no evidence in favour of TBI-based RIC regimens over chemotherapy- only conditioning or vice versa [81, 94]. Therefore, the RIC regimen used should be one the centre is familiar with.

#### Donor selection and graft source

Similar to the other main lymphoma entities haplotransplantation can be safely and successfully performed in MCL using the post-transplant cyclophosphamide (PTCy) platform [91], implying that a suitable donor should be readily available for almost every patient [95, 96]. However, HLA-matching still matters if uniform GVHD prophylaxis conditions are observed [97]. Therefore, general guidelines for donor selection should be followed, currently recommending a hierarchical selection in the order MSD, MUD, 1-antigen-mismatched unrelated donor (MMUD) or haplo-identical donor (PTCy platform), cord blood [96].

Peripheral blood and bone marrow hematopoietic cells are both acceptable sources of donor stem cells from matched sibling and matched unrelated donors [81]. Despite better engraftment [91], it remains unclear whether peripheral blood should be preferred for haplotype transplants.

#### GVHD prophylaxis

There is no GVHD prophylaxis regimen specific for MCL. GVHD prevention strategies should be selected based on the donor type and conditioning regimen employed and the transplant centres local expertise, taking into account contemporary developments in evidence-based standards.

**Panel 11. Recommendations for allo-HCT transplantation modalities**

- **Conditioning**
- RIC should be preferred.
- There is no evidence for the superiority of any specific regimen; the RIC regimen used should be one that the centre is familiar with.
- **Donor and stem cell source**
- For donor selection, general guidelines for donor selection should be followed.
- Currently, this means MRD > MUD > MMUD/MMRD > CB.
- Both peripheral blood and bone marrow are acceptable sources of hematopoietic cell grafts from matched sibling and matched unrelated donors.
- **GVHD prevention**
- GVHD prevention strategies should be selected based on the donor type and conditioning regimen employed and the transplant centres local expertise.

#### Post-allo-HCT management

There is no evidence supporting antibody- or BTKi-based maintenance after allo-HCT in MCL.

Pre-emptive immune modulation by donor lymphocyte infusions (DLI) based on post-transplant longitudinal monitoring of chimerism as MRD surrogate has been investigated only for alemtuzumab-containing platforms in MCL, without showing a clear-cut benefit [98]. However, since MCL is a DLI-sensitive disease [99]. and alternative immune-inert therapeutic options for pre-emptive use in MCL, such as BTKi and CD20 antibodies, are available, it seems reasonable to follow individual centres’ strategies of post-transplant MRD surveillance.

General posttransplant surveillance, supportive care and infection prophylaxis should follow general guidelines [77].

## Unanswered questions and research priorities

Since our preceding recommendations paper on cellular therapy in MCL [3], additional innovations have entered routine MCL care, e.g. extension of the acalabrutinib and lisocel labels to MCL, and ibrutinib in combination with chemoimmunotherapy to the first line. This illustrates the dynamic speed of transformation in the MCL management landscape—with obvious impact on the role and use of cellular therapies in this entity, necessitating steady adaptation of clinical practice recommendations to the evolving authorization framework and scientific state-of-the-art.

Many recommendations contained in this paper rely on expert opinion rather than evidence, thus pinpointing the current most urgent research priorities, such as defining the optimum timing of CART and allo-HCT in the different risk settings, and the role of second CART treatments and bispecific antibodies in MCL. A particular matter of debate among the experts involved in this project was defining the role of first-line auto-HCT in the BTKi era, implying that this is a research topic of urgent priority. With novel pathway inhibitors, bispecific antibodies, antibody-drug compounds and their combinations, but also innovative CART platforms *ante portas* [29, 100], the role of auto-HCT is expected to fade, while allo-HCT will move further downstream in the treatment algorithm. Nevertheless, allo-HCT remains the only modality with proven curative potential in MCL to date, making further optimization of the efficacy-safety profile of this procedure an important research priority.

## References

[1] Robinson S, Dreger P, Caballero D, et al. The EBMT/EMCL consensus project on the role of autologous and allogeneic stem cell transplantation in mantle cell lymphoma. Leukemia. 2015;29:464–73. 25034148 10.1038/leu.2014.223

[2] Munshi PN, Hamadani M, Kumar A, et al. ASTCT, CIBMTR, and EBMT clinical practice recommendations for transplant and cellular therapies in mantle cell lymphoma. Bone Marrow Transpl. 2021;56:2911–21. 10.1038/s41409-021-01288-9 PMC8639670 34413469

[3] Dreger P, Ahmed S, Bazarbachi A, et al. How we treat mantle cell lymphoma with cellular therapy in 2025: the European and American perspectives. Bone Marrow Transpl. 2025;60:759–68. 10.1038/s41409-025-02599-x PMC12151868 40229536

[4] Munshi PN, Hamadani M, Kumar A, et al. American Society of Transplantation and Cellular Therapy, Center of International Blood and Marrow Transplant Research, and European Society for Blood and Marrow Transplantation Clinical Practice Recommendations for Transplantation and Cellular Therapies in Mantle Cell Lymphoma. Transpl Cell Ther. 2021;27:720–8. 10.1016/j.jtct.2021.03.001 PMC8447221 34452722

[5] Yakoub-Agha I, Greco R, Onida F, et al. Practice harmonization workshops of EBMT: an expert-based approach to generate practical and contemporary guidelines within the arena of hematopoietic cell transplantation and cellular therapy. Bone Marrow Transpl. 2023;58:696–700. 10.1038/s41409-023-01958-w PMC10247369 36973515

[6] Eyre TA, Bishton MJ, McCulloch R, et al. Diagnosis and management of mantle cell lymphoma: A British Society for Haematology Guideline. Br J Haematol. 2024;204:108–26. 37880821 10.1111/bjh.19131

[7] Jerkeman M, Aurer I, Campo E, et al. EHA-EU MCL network guidelines for diagnosis and treatment of mantle cell lymphoma. Hemasphere. 2025;9:e70233. 41132246 10.1002/hem3.70233 PMC12541557

[8] Eyre TA, Cwynarski K, d’Amore F, et al. Lymphomas: ESMO Clinical Practice Guideline for diagnosis, treatment and follow-up. Ann Oncol. 2025;36:1263–84. 40774601 10.1016/j.annonc.2025.07.014

[9] Eskelund CW, Dahl C, Hansen JW, et al. TP53 mutations identify younger mantle cell lymphoma patients who do not benefit from intensive chemoimmunotherapy. Blood. 2017;130:1903–10. 28819011 10.1182/blood-2017-04-779736

[10] Ferrero S, Rossi D, Rinaldi A, et al. KMT2D mutations and TP53 disruptions are poor prognostic biomarkers in mantle cell lymphoma receiving high-dose therapy: a FIL study. Haematologica. 2020;105:1604–12. 31537689 10.3324/haematol.2018.214056 PMC7271566

[11] Greiner TC, Moynihan MJ, Chan WC, et al. p53 mutations in mantle cell lymphoma are associated with variant cytology and predict a poor prognosis. Blood. 1996;87:4302–10. 8639789

[12] Scheubeck G, Jiang L, Hermine O, et al. Clinical outcome of mantle cell lymphoma patients with high-risk disease (high-risk MIPI-c or high p53 expression). Leukemia. 2023;37:1887–94. 37495776 10.1038/s41375-023-01977-y PMC10457193

[13] Eskelund CW, Kolstad A, Jerkeman M, et al. 15-year follow-up of the second Nordic mantle cell lymphoma trial (MCL2): prolonged remissions without survival plateau. Br J Haematol. 2016;175:410–8. 27378674 10.1111/bjh.14241

[14] Hoster E, Rosenwald A, Berger F, et al. Prognostic value of Ki-67 index, cytology, and growth pattern in mantle-cell lymphoma: results from randomized trials of the European Mantle Cell Lymphoma Network. J Clin Oncol. 2016;34:1386–94. 26926679 10.1200/JCO.2015.63.8387

[15] Rule S, Dreyling M, Goy A, et al. Outcomes in 370 patients with mantle cell lymphoma treated with ibrutinib: a pooled analysis from three open-label studies. Br J Haematol. 2017;179:430–9. 28832957 10.1111/bjh.14870 PMC5912680

[16] Lewis DJ, Jerkeman M, Sorrell L, et al. Ibrutinib and rituximab versus immunochemotherapy in patients with previously untreated mantle cell lymphoma (ENRICH): a randomised, open-label, phase 2/3 superiority trial. Lancet. 2025;406:1953–68. 41052510 10.1016/S0140-6736(25)01432-1 PMC12549478

[17] Forsgren E, Jorgensen RRK, Bentzen H, et al. Ibrutinib, lenalidomide, and rituximab in relapsed mantle cell lymphoma: long-term follow-up of the Nordic lymphoma group MCL6 Philemon trial. Hemasphere. 2025;9:e70101. 40104044 10.1002/hem3.70101 PMC11914894

[18] Visco C, Tisi MC, Evangelista A, et al. Time to progression of mantle cell lymphoma after high-dose cytarabine-based regimens defines patients risk for death. Br J Haematol. 2019;185:940–4. 30407625 10.1111/bjh.15643

[19] Eskelund CW, Dimopoulos K, Kolstad A, et al. Detailed long-term follow-up of patients who relapsed after the Nordic mantle cell lymphoma trials: MCL2 and MCL3. Hemasphere. 2021;5:e510. 33364550 10.1097/HS9.0000000000000510 PMC7755521

[20] Sarkozy C, Chartier L, Ribrag V, et al. Validation of POD24 as a robust early clinical indicator of poor survival in mantle cell lymphoma from 1280 patients on clinical trials, a LYSA study. Blood. Cancer J. 2025;15:78. 10.1038/s41408-025-01241-9 PMC12022242 40274771

[21] Rezazadeh A, Pruett J, Detzner A, et al. Immunoglobulin high-throughput sequencing (Ig-HTS) minimal residual disease (MRD) analysis is an effective surveillance tool in patients with mantle cell lymphoma. Clin Lymphoma Myeloma Leuk. 2024;24:254–9. 38195321 10.1016/j.clml.2023.12.006

[22] Fenske TS, Wang XV, Till BG, et al. Lack of benefit of autologous hematopoietic cell transplantation (auto-HCT) in mantle cell lymphoma patients in first complete remission with undetectable minimal residual disease (uMRD): initial report from the ECOG-ACRIN EA4151 Phase 3. Blood. 2024;144:LBA–6.

[23] Alaggio R, Amador C, Anagnostopoulos I, et al. The 5th edition of the World Health Organization Classification of Haematolymphoid Tumours: Lymphoid Neoplasms. Leukemia. 2022;36:1703–19. 35732829 10.1038/s41375-022-01620-2 PMC9214472

[24] Wilson MR, Barrett A, Cheah CY, Eyre TA. How I manage mantle cell lymphoma: indolent versus aggressive disease. Br J Haematol. 2023;201:185–98. 36807902 10.1111/bjh.18697

[25] Dreyling M, Doorduijn J, Gine E, et al. Ibrutinib combined with immunochemotherapy with or without autologous stem-cell transplantation versus immunochemotherapy and autologous stem-cell transplantation in previously untreated patients with mantle cell lymphoma (TRIANGLE): a three-arm, randomised, open-label, phase 3 superiority trial of the European Mantle Cell Lymphoma Network. Lancet. 2024;403:2293–306. 38705160 10.1016/S0140-6736(24)00184-3

[26] Eyre TA, Cheah CY, Sarkozy C, Kumar A, Le GS. Mantle cell lymphoma: optimal treatment with Bruton tyrosine kinase-targeted approaches. J Clin Oncol. 2025;43:2300–10. 40440566 10.1200/JCO-25-00146

[27] Greco R, Ruggeri A, McLornan DP, et al. Indications for haematopoietic cell transplantation and CAR-T for haematological diseases, solid tumours and immune disorders: 2025 EBMT practice recommendations. Bone Marrow Transpl. 2025;60:1499–525. 10.1038/s41409-025-02701-3 PMC12583170 40926035

[28] van Meerten T, Kersten MJ, Iacoboni G et al. Brexucabtagene autoleucel for BTKi-naive relapsed/refractory mantle cell lymphoma: primary analysis of ZUMA-2 Cohort 3. Blood. 2025;147:1302–1314. 10.1182/blood.2025029734 PMC13014085 41160777

[29] Shah NN, Colina AS, Johnson BD et al. Phase I/II study of adaptive manufactured lentiviral anti-CD20/anti-CD19 chimeric antigen receptor T cells for relapsed, refractory mantle cell lymphoma. J Clin Oncol. 2025;43:2285–2295. 10.1200/JCO-24-02158 40163793

[30] Schubert ML, Dietrich S, Stilgenbauer S, et al. Feasibility and safety of CD19 CAR T cell treatment for B-cell lymphoma relapse after allogeneic hematopoietic stem cell transplantation. Biol Blood Marrow Transpl. 2020;26:1575–80. 10.1016/j.bbmt.2020.04.025 32422254

[31] Kharfan-Dabaja MA, Mohty R, Easwar N, et al. Chimeric antigen receptor T cell therapy in octogenarians with B cell lymphoma: a real-world US multicenter collaborative study. Bone Marrow Transpl. 2025;60:632–9. 10.1038/s41409-025-02541-1 40025178

[32] Ahmed N, Thiruvengadam S, Hamadani M, et al. Real-world outcomes of brexucabtagene autoleucel for relapsed or refractory mantle cell lymphoma: a CIBMTR analysis. Blood Adv. 2025;9:5382–96. 40706035 10.1182/bloodadvances.2024015014 PMC12597645

[33] Dreger P, Corradini P, Gribben JG, et al. CD19-directed CAR-T cells as first salvage therapy for large B-cell lymphoma: towards a rational approach. Lancet Haematol. 2023;10:e1006–e1015. 38030311 10.1016/S2352-3026(23)00307-1

[34] Jacobson CA, Locke FL, Ma L, et al. Real-world evidence of axicabtagene ciloleucel for the treatment of large B-cell lymphoma in the United States. Transpl Cell Ther. 2022;28:581.e1–581.e8. 10.1016/j.jtct.2022.05.026 PMC9427701 35609867

[35] Beitinjaneh A, Gupta S, Furqan F et al. Real-world effectiveness and safety outcomes by age, comorbidities, and frailty among relapsed or refractory (R/R) Mantle Cell Lymphoma (MCL) patients treated with brexucabtagene autoleucel (brexu-cel). Blood. 2025;144:3606.

[36] Lin RJ, Kim SJ, Brown S, et al. Prospective geriatric assessment and geriatric consultation in CAR T-cell therapy for older patients with lymphoma. Blood Adv. 2023;7:3501–5. 37078703 10.1182/bloodadvances.2023010003 PMC10362256

[37] Yates SJ, Cursio JF, Artz A, et al. Optimization of older adults by a geriatric assessment-guided multidisciplinary clinic before CAR T-cell therapy. Blood Adv. 2024;8:3785–97. 38810262 10.1182/bloodadvances.2024012727 PMC11298834

[38] Wang M, Muñoz J, Goy A, et al. KTE-X19 CAR T-cell therapy in relapsed or refractory mantle-cell lymphoma. N Engl J Med. 2020;382:1331–42. 32242358 10.1056/NEJMoa1914347 PMC7731441

[39] Wang Y, Jain P, Locke FL, et al. Brexucabtagene autoleucel for relapsed or refractory mantle cell lymphoma in standard-of-care practice: results from the US Lymphoma CAR T Consortium. J Clin Oncol. 2023;41:2594–606. 36753699 10.1200/JCO.22.01797 PMC10489553

[40] O’Reilly MA, Wilson W, Burns D, et al. Brexucabtagene autoleucel for relapsed or refractory mantle cell lymphoma in the United Kingdom: a real-world intention-to-treat analysis. Hemasphere. 2024;8:e87. 38873532 10.1002/hem3.87 PMC11170269

[41] Simon L, Vucinic V, Rejeski K, et al. Results of brexucabtagene-autoleucel for patients with relapsed/refractory mantle cell lymphoma in the routine setting in Germany and Switzerland. Bone Marrow Transplant. 2026;61:480–483. 10.1038/s41409-025-02789-7 PMC13056568 41545665

[42] Locke FL, Filosto S, Chou J, et al. Impact of tumor microenvironment on efficacy of anti-CD19 CAR T cell therapy, chemotherapy and transplant in large B cell lymphoma. Nat Med. 2024;30:507–18. 38233586 10.1038/s41591-023-02754-1 PMC10878966

[43] Ahmed G, Alsouqi A, Szabo A, et al. CAR T-cell therapy in mantle cell lymphoma with secondary CNS involvement: a multicenter experience. Blood Adv. 2024;8:3528–31. 38701405 10.1182/bloodadvances.2023012255 PMC11261102

[44] Rejeski K, Wang Y, Albanyan O, et al. The CAR-HEMATOTOX score identifies patients at high risk for hematological toxicity, infectious complications, and poor treatment outcomes following brexucabtagene autoleucel for relapsed or refractory MCL. Am J Hematol. 2023;98:1699–710. 37584447 10.1002/ajh.27056 PMC10659121

[45] Frenking JH, Zhou X, Wagner V et al. EASIX-guided risk stratification for complications and outcome after CAR T-cell therapy with ide-cel in relapsed/refractory multiple myeloma. J Immunother Cancer 2024;12:e009220. 10.1136/jitc-2024-009220 PMC11459298 39379098

[46] Herbaux C, Bret C, Bachy E, et al. Brexucabtagene autoleucel in relapsed or refractory mantle cell lymphoma, intention-to-treat use in the DESCAR-T registry. Haematologica. 2024;109:3745–50. 38899352 10.3324/haematol.2023.284786 PMC11532701

[47] Wang M, Siddiqi T, Gordon LI, et al. Lisocabtagene maraleucel in relapsed/refractory mantle cell lymphoma: primary analysis of the mantle cell lymphoma cohort from TRANSCEND NHL 001, a phase I Multicenter Seamless Design Study. J Clin Oncol. 2024;42:1146–57. 38072625 10.1200/JCO.23.02214 PMC11741176

[48] Wang ML, Jurczak W, Zinzani PL, et al. Pirtobrutinib a covalent Bruton tyrosine kinase inhibitor pretreated mantle-cell lymphoma. J Clin Oncol. 2023;41:3988–97. 37192437 10.1200/JCO.23.00562 PMC10461952

[49] Wang M, Muñoz J, Goy A, et al. Three-year follow-up of KTE-X19 in patients with relapsed/refractory mantle cell lymphoma, including high-risk subgroups, in the ZUMA-2 study. J Clin Oncol. 2023;41:555–67. 35658525 10.1200/JCO.21.02370 PMC9870225

[50] Iacoboni G, Navarro V, Martin-Lopez AA, et al. Recent bendamustine treatment before apheresis has a negative impact on outcomes in patients with large B-cell lymphoma receiving chimeric antigen receptor T-cell therapy. J Clin Oncol. 2024;42:205–17. 37874957 10.1200/JCO.23.01097

[51] Camus V, Houot R, Brisou G, et al. Outcome of patients with large B-cell lymphoma treated with tafasitamab plus lenalidomide either before or after CAR T-cell therapy. Blood Adv. 2024;8:5371–81. 39163620 10.1182/bloodadvances.2024013726 PMC11568786

[52] Le Gouill S, Morschhauser F, Chiron D, et al. Ibrutinib, obinutuzumab, and venetoclax in relapsed and untreated patients with mantle cell lymphoma: a phase 1/2 trial. B. 2021;137:877–87. 10.1182/blood.2020008727 33181832

[53] Wang M, Jurczak W, Trneny M, et al. Ibrutinib plus venetoclax in relapsed or refractory mantle cell lymphoma (SYMPATICO): a multicentre, randomised, double-blind, placebo-controlled, phase 3 study. Lancet Oncol. 2025;26:200–13. 39914418 10.1016/S1470-2045(24)00682-X

[54] Phillips TJ, Carlo-Stella C, Morschhauser F, et al. Glofitamab in relapsed/refractory mantle cell lymphoma: results from a Phase I/II study. J Clin Oncol. 2025;43:318–28. 39365960 10.1200/JCO.23.02470 PMC11771347

[55] Fraietta JA, Beckwith KA, Patel PR, et al. Ibrutinib enhances chimeric antigen receptor T-cell engraftment and efficacy in leukemia. Blood. 2016;127:1117–27. 26813675 10.1182/blood-2015-11-679134 PMC4778162

[56] Minson A, Hamad N, Cheah CY, et al. CAR T cells and time-limited ibrutinib as treatment for relapsed/refractory mantle cell lymphoma: the phase 2 TARMAC study. B. 2024;143:673–84. 10.1182/blood.2023021306 37883795

[57] Hayden PJ, Roddie C, Bader P, et al. Management of adults and children receiving CAR T-cell therapy: 2021 best practice recommendations of the European Society for Blood and Marrow Transplantation (EBMT) and the Joint Accreditation Committee of ISCT and EBMT (JACIE) and the European Haematology Association (EHA). Ann Oncol. 2022;33:259–75. 34923107 10.1016/j.annonc.2021.12.003

[58] Cordas Dos Santos DM, Tix T, Shouval R, et al. A systematic review and meta-analysis of nonrelapse mortality after CAR T cell therapy. Nat Med. 2024;30:2667–78. 38977912 10.1038/s41591-024-03084-6 PMC11765209

[59] Bailly C, Carlier T, Berriolo-Riedinger A, et al. Prognostic value of FDG-PET in patients with mantle cell lymphoma: results from the LyMa-PET Project. Haematologica. 2020;105:e33–e36. 31371411 10.3324/haematol.2019.223016 PMC6939504

[60] Deschenes-Simard X, Bromberg M, Devlin SM, et al. Comparative real-world outcomes of CD19-directed CAR T-cell therapies in large B-cell lymphoma. Blood Adv. 2025;9:5571–84. 40815804 10.1182/bloodadvances.2025016778 PMC12615284

[61] Dreyling M, Lenz G, Hoster E, et al. Early consolidation by myeloablative radiochemotherapy followed by autologous stem cell transplantation in first remission significantly prolongs progression-free survival in mantle-cell lymphoma: results of a prospective randomized trial of the European MCL Network. Blood. 2005;105:2677–84. 15591112 10.1182/blood-2004-10-3883

[62] Geisler CH, Kolstad A, Laurell A, et al. Long-term progression-free survival of mantle cell lymphoma following intensive front-line immunochemotherapy with in vivo-purged stem cell rescue: a non-randomized phase-II multicenter study by the Nordic Lymphoma Group. Blood. 2008;112:2687–93. 18625886 10.1182/blood-2008-03-147025 PMC2556606

[63] Hermine O, Hoster E, Walewski J, et al. Addition of high-dose cytarabine to immunochemotherapy before autologous stem-cell transplantation in patients aged 65 years or younger with mantle cell lymphoma (MCL Younger): a randomised, open-label, phase 3 trial of the European Mantle Cell Lymphoma Network. Lancet. 2016;388:565–75. 27313086 10.1016/S0140-6736(16)00739-X

[64] Dreyling M, Doorduijn JK, Gine E, et al. Role of autologous stem cell transplantation in the context of ibrutinib-containing first-line treatment in younger patients with mantle cell lymphoma: results from the randomized triangle trial by the European MCL Network. Blood. 2024;144:240.

[65] Ladetto M, Gutmair K, Doorduijn JK, et al. Impact of Rituximab maintenance added to ibrutinib-containing regimens with and without ASCT in younger, previously untreated MCL patients: an analysis of the triangle data embedded in the multiply project. Blood. 2024;144:237.38990538

[66] Ragaini S, Gutmair K, Moia R et al. Prognostic impact of specific IGHV rearrangements in younger untreated Mantle Cell Lymphoma patients is independent of p53 alterations and may be modified by ibrutinib-based regimens: Insights from the TRIANGLE trial within the MULTIPLY project. Blood 2025;146:5299.

[67] Martin P, Cohen JB, Wang M et al. Treatment outcomes and roles of transplantation and maintenance rituximab in patients with previously untreated mantle cell lymphoma: results from large real-world cohorts. J Clin Oncol. 2023;41:541–554. 10.1200/JCO.21.02698 PMC9870229 35763708

[68] Houot R, Bachy E, Cartron G et al. Axicabtagene ciloleucel in large B cell lymphoma ineligible for autologous stem cell transplantation: the phase 2 ALYCANTE trial. Nat Med. 2023;29:2593–2601 10.1038/s41591-023-02572-5 PMC10579056 37710005

[69] Sehgal A, Hoda D, Riedell PA, et al. Lisocabtagene maraleucel as second-line therapy in adults with relapsed or refractory large B-cell lymphoma who were not intended for haematopoietic stem cell transplantation (PILOT): an open-label, phase 2 study. Lancet Oncol. 2022;23:1066–77. 35839786 10.1016/S1470-2045(22)00339-4

[70] Jantunen E, Canals C, Attal M, et al. Autologous stem-cell transplantation in patients with mantle cell lymphoma beyond 65 years of age: a study from the European Group for Blood and Marrow Transplantation (EBMT). Ann Oncol. 2012;23:166–71. 21467125 10.1093/annonc/mdr035

[71] Zoellner AK, Unterhalt M, Stilgenbauer S, et al. Long-term survival of patients with mantle cell lymphoma after autologous haematopoietic stem-cell transplantation in first remission: a post-hoc analysis of an open-label, multicentre, randomised, phase 3 trial. Lancet Haematol. 2021;8:e648–e657. 34450102 10.1016/S2352-3026(21)00195-2

[72] Tseng YD, Stevenson PA, Cassaday RD, et al. Total Body Irradiation is safe and similarly effective as chemotherapy-only conditioning in autologous stem cell transplantation for mantle cell lymphoma. Biol Blood Marrow Transpl. 2018;24:282–7. 10.1016/j.bbmt.2017.10.029 29061536

[73] Peyrade F, Jardin F, Thieblemont C, et al. Attenuated immunochemotherapy regimen (R-miniCHOP) in elderly patients older than 80 years with diffuse large B-cell lymphoma: a multicentre, single-arm, phase 2 trial. Lancet Oncol. 2011;12:460–8. 21482186 10.1016/S1470-2045(11)70069-9

[74] Chen YB, Lane AA, Logan BR, et al. Impact of conditioning regimen on outcomes for patients with lymphoma undergoing high-dose therapy with autologous hematopoietic cell transplantation. Biol Blood Marrow Transpl. 2015;21:1046–53. 10.1016/j.bbmt.2015.02.005 PMC4426014 25687795

[75] Sarkozy C, Thieblemont C, Oberic L, et al. Long-term follow-up of rituximab maintenance in young patients with mantle-cell lymphoma included in the LYMA trial: A LYSA Study. J Clin Oncol. 2024;42:769–73. 38109684 10.1200/JCO.23.01586

[76] Andersen NS, Pedersen LB, Laurell A, et al. Pre-emptive treatment with rituximab of molecular relapse after autologous stem cell transplantation in mantle cell lymphoma. J Clin Oncol. 2009;27:4365–70. 19652064 10.1200/JCO.2008.21.3116

[77] Sureda A, Corbacioglu S, Greco R, Kröger N, and Carreras E. The EBMT handbook. hematopoietic cell transplantation and cellular therapies. Springer. Ref Type: Online Source. 2026 https://www.ebmt.org/education/ebmt-handbook. 39437029

[78] Dreger P, Michallet M, Bosman P, et al. Ibrutinib for bridging to allogeneic hematopoietic cell transplantation in patients with chronic lymphocytic leukemia or mantle cell lymphoma: a study by the EBMT Chronic Malignancies and Lymphoma Working Parties. Bone Marrow Transpl. 2019;54:44–52. 10.1038/s41409-018-0207-4 29728701

[79] McCulloch R, Visco C, Eyre TA, et al. Efficacy of R-BAC in relapsed, refractory mantle cell lymphoma post BTK inhibitor therapy. Br J Haematol. 2020;189:684–8. 32011729 10.1111/bjh.16416

[80] Liebers N, Boumendil A, Finel H, et al. Brexucabtagene autoleucel versus allogeneic hematopoietic cell transplantation in relapsed and refractory mantle cell lymphoma. Blood Cancer Discov. 2025;6:182–90. 39913291 10.1158/2643-3230.BCD-24-0178 PMC12050943

[81] Robinson SP, Boumendil A, Finel H, et al. Long-term outcome analysis of reduced-intensity allogeneic stem cell transplantation in patients with mantle cell lymphoma: a retrospective study from the EBMT Lymphoma Working Party. Bone Marrow Transpl. 2018;53:617–24. 10.1038/s41409-017-0067-3 29335632

[82] Epstein-Peterson ZD, Lionel AC, Joseph A, et al. Treatment and outcomes of progression of disease post-CAR T-cell therapy in mantle cell lymphoma: a multicenter analysis. Blood Adv. 2025;9:5654–62. 40763269 10.1182/bloodadvances.2025016315 PMC12848353

[83] Zurko J, Ramdial J, Shadman M, et al. Allogeneic transplant following CAR T-cell therapy for large B-cell lymphoma. Haematologica. 2023;108:98–109. 35833303 10.3324/haematol.2022.281242 PMC9827150

[84] Derigs P, Bethge WA, Kramer I, et al. Long-term survivors after failure of CAR-T cell therapy for large B-cell lymphoma: a role for allogeneic hematopoietic cell transplantation? A GLA/DRST analysis. Transpl Cell Ther. 2023;29:750–6. 10.1016/j.jtct.2023.09.008 37709204

[85] Kyriakou C, Boumendil A, Finel H, et al. The impact of advanced patient age on mortality after allogeneic hematopoietic cell transplantation for non-Hodgkin’s lymphoma: a retrospective study by the EBMT Lymphoma Working Party. Biol Blood Marrow Transpl. 2019;25:86–93. 10.1016/j.bbmt.2018.08.025 30219698

[86] Shah NN, Ahn KW, Litovich C, et al. Allogeneic transplantation in elderly patients >/=65 years with non-Hodgkin lymphoma: a time-trend analysis. Blood. Cancer J. 2019;9:97. 10.1038/s41408-019-0261-1 PMC6890709 31796726

[87] Lin RJ, Ho C, Hilden PD, et al. Allogeneic haematopoietic cell transplantation impacts on outcomes of mantle cell lymphoma with TP53 alterations. Br J Haematol. 2019;184:1006–10. 30537212 10.1111/bjh.15721 PMC6399037

[88] Hamadani M, Saber W, Ahn KW, et al. Allogeneic hematopoietic cell transplantation for chemotherapy-unresponsive mantle cell lymphoma: a cohort analysis from the Centre for International Blood and Marrow Transplant Research. Biol Blood Marrow Transpl. 2013;19:625–31. 10.1016/j.bbmt.2013.01.009 PMC3640440 23333532

[89] Barone A, de PC, Stella F, et al. Allogeneic transplantation after failure of chimeric antigen receptor-T cells and exposure to bispecific antibodies: feasibility, safety and survival outcomes. Br J Haematol. 2025;207:956–64. 40695357 10.1111/bjh.70010 PMC12436228

[90] Tuve S, Gayoso J, Scheid C, et al. Haploidentical bone marrow transplantation with post-grafting cyclophosphamide: multicenter experience with an alternative salvage strategy. Leukemia. 2011;25:880–3. 21311556 10.1038/leu.2011.11

[91] Bazarbachi A, Boumendil A, Finel H, et al. Influence of donor type, stem cell source and conditioning on outcomes after haploidentical transplant for lymphoma - a LWP-EBMT study. Br J Haematol. 2020;188:745–56. 31498883 10.1111/bjh.16182

[92] Kharfan-Dabaja MA, Reljic T, El-Asmar J, et al. Reduced-intensity or myeloablative allogeneic hematopoietic cell transplantation for mantle cell lymphoma: a systematic review. Future Oncol. 2016;12:2631–42. 27381652 10.2217/fon-2016-0146

[93] Ghosh N, Ahmed S, Ahn KW, et al. Association of reduced-intensity conditioning regimens with overall survival among patients with non-Hodgkin lymphoma undergoing allogeneic transplant. JAMA Oncol. 2020;6:1011–8. 32496525 10.1001/jamaoncol.2020.1278 PMC7273311

[94] Tessoulin B, Ceballos P, Chevallier P, et al. Allogeneic stem cell transplantation for patients with mantle cell lymphoma who failed autologous stem cell transplantation: a national survey of the SFGM-TC. Bone Marrow Transpl. 2016;51:1184–90. 10.1038/bmt.2016.102 27111043

[95] Selberg L, Stadtherr P, Dietrich S, et al. The impact of allogeneic hematopoietic cell transplantation on the mortality of poor-risk non-Hodgkin lymphoma: an intent-to-transplant analysis. Bone Marrow Transpl. 2021;56:30–37. 10.1038/s41409-020-0976-4 32555407

[96] Jimenez Jimenez AM, Spellman SR, Politikos I, et al. Allogeneic hematopoietic cell donor selection: contemporary guidelines from the NMDP/CIBMTR. Transpl Cell Ther. 2025;31:973–88. 10.1016/j.jtct.2025.07.004 PMC12344661 40619101

[97] Mussetti A, Kanate AS, Wang T, et al. Haploidentical versus matched unrelated donor transplants using post-transplant cyclophosphamide for lymphomas. Transpl Cell Ther. 2022;29:184.e1–184.e9. 10.1016/j.jtct.2022.11.028 PMC10316698 36577482

[98] Cook G, Smith GM, Kirkland K, et al. Outcome following reduced intensity allogeneic stem cell transplantation (RIC AlloSCT) for relapsed and refractory mantle-cell lymphoma (MCL): a study of the British Society for Blood and Marrow Transplantation. Biol Blood Marrow Transpl. 2010;16:1419–27. 10.1016/j.bbmt.2010.04.006 20399879

[99] Robinson S, Boumendil A, Finel H, et al. Donor lymphocyte infusions induce durable responses in patients with follicular, mantle and T cell lymphomas relapsing after an alloSCT. An EBMT-LWP study. Hematol Oncol. 2020;37:316–7.

[100] Phillips T, Chen L, Barnhizer T, et al. Interim analysis of the phase II study of glofitamab, lenaliomide and venetoclax (GLOVe) in untreated patients w/ high-risk mantle cell lymphoma. response and safety outcomes after the completion of stage 1 of 2 enrollment. Blood. 2025;146:833.

[101] Stella F, Chiappella A, Magni M, et al. Brexucabtagene autoleucel in vivo expansion and BTKi refractoriness have a negative influence on progression-free survival in mantle cell lymphoma: results from the CART-SIE study. Br J Haematol. 2025;206:644–51. 39710966 10.1111/bjh.19961 PMC11829141

